# Efficacy and safety of onabotulinumtoxinA in the treatment of medication overuse headache: a systematic review

**DOI:** 10.3389/fneur.2024.1453183

**Published:** 2024-09-20

**Authors:** Hui Lang, Cheng Peng, Kongyuan Wu, Xiwen Chen, Xin Jiang, Li He, Ning Chen

**Affiliations:** ^1^Department of Neurology, West China Hospital of Sichuan University, Chengdu, China; ^2^Department of Neurology, The Second Affiliated Hospital of Chengdu Medical College, China National Nuclear Corporation 416 Hospital, Chengdu, Sichuan, China

**Keywords:** medication overuse headache, onabotulinumtoxinA, systematic review, headache, treatment

## Abstract

**Purpose:**

Medication overuse headache (MOH) is a chronic headache caused by regular overuse of medications. OnabotulinumtoxinA (BoNTA) is used for preventive treatment of MOH. However, its efficacy and safety remain controversial.

**Methods:**

Seven online databases (Cochrane Library, Embase, Medline, PubMed, China National Knowledge Infrastructure, Wanfang data, and Chinese BioMedical Literature Database) were searched for relevant articles published between January 2002 and March 2024. We included randomized controlled trials (RCTs) and cohort studies on the treatment of MOH using BoNTA versus a placebo or other active treatments.

**Results:**

We retrieved 487 articles in the database search. Of these, four eligible RCTs were identified after detailed screening. A total of 1,259 patients with MOH (622 patients treated with BoNTA, 607 with placebo, and 30 with topiramate) were included in the four RCTs. We found that BoNTA significantly reduced headache frequency compared with placebo (mean difference, 1.89; 95% confidence interval (CI), 1.11–2.67; *I*^2^ = 0%; *p* < 0.001). There was no significant difference between BoNTA and the placebo in terms of secondary outcomes, which included reductions in acute medication intake (MD, 1.30; 95% CI, −1.18–3.78; *I*^2^ = 0%; *p* = 0.30), Migraine Disability Assessment questionnaire scores (MIDAS, MD, −4.04; 95% CI, −29.36–21.28; *I*^2^ = 0%; *p* = 0.75), and Headache Impact Test scores (HIT-6, MD, 0.03; 95% CI, −1.77–1.83; *I*^2^ = 0%; *p* = 0.97). BoNTA was more likely to cause adverse events (OR, 1.87; 95% CI, 1.45–2.42; *I*^2^ = 0%; *p* < 0.001) than placebo.

**Conclusion:**

The results of this study show that BoNTA reduces headache frequency and is effective for the treatment of MOH.

**Systematic review registration:**

https://www.crd.york.ac.uk/prospero/, identifier CRD42022315845.

## 1 Introduction

Medication overuse headache (MOH) is that occur 15 or more days per month in patients with a primary headache (such as migraine and tension-type headache) due to regular overuse of acute or symptomatic medication for >3 months (1). Overuse of medications such as ergotamine, triptan, non-opioid analgesic (acetaminophen, nonsteroidal anti-inflammatory drugs), opioids, combination-analgesic and butalbital (≥15 days for acetaminophen and nonsteroidal anti-inflammatory drugs, and > 10 days for the rest) leads to MOH (1).

There is an increased susceptibility to MOH in people who take medication (tranquillizers, aspirin, ibuprofen, opioids), people with migraine, people with anxiety and women (2). Approximately 11–70% of adult individuals with chronic migraine (CM) take acute symptomatic medications excessively, which increases the risk of developing MOH (3). The global prevalence of MOH in adults is approximately 0.5–2.0% in adults worldwide (4). MOH is a chronic and disabling disease that is often accompanied by mood disorders, comorbidities, and its treatment burden is three times that of episodic migraine. In addition, MOH places considerable mental pressure and financial burden on patients (5). Currently, the common clinical treatment options for MOH are health education, drug withdrawal, and prophylactic therapy. Preventive treatments include both biobehavioral prevention and drug prevention. Biobehavioral prevention includes avoidance of trigger factors, stress management, and cognitive behavioral management (6). Preventive drugs for MOH include topiramate, onabotulinumtoxinA (BoNTA), monoclonal antibodies against calcitonin gene-related peptide (CGRP) or CGRP receptor, and valproic acid (6, 7).

Botulinum toxins are a group of neurotoxins produced by *Clostridium botulinum*. In clinical practice, botulinum toxins can be used for the treatment of movement disorders (such as blepharospasm, cervical dystonia, upper and lower limb spasticity) and autonomic dysfunction (hypersalivation, hyperhidrosis) (8, 9). In neurology, BoNTA is also used to treat CM. BoNTA inhibits the release of nociceptive mediators (glutamate, substance P, CGRP), which can suppress neurogenic inflammation and peripheral sensitization of nociceptive nerve fibers (10, 11).

Studies have confirmed the efficacy and safety of BoNTA for the treatment of CM. In clinical practice, BoNTA can be used as a prophylactic treatment for MOH because of its inhibitory effect on the release of nociceptive mediators (11). However, its efficacy and safety for the treatment of MOH remain controversial. The authors of one study reported that BoNTA could significantly reduce the number of headache and medication intake days in patients with MOH, thereby improving their quality of life (12). Other researchers concluded that BoNTA has no effect on MOH (13). Therefore, we performed a systematic review to investigate whether BoNTA is effective and safe for the treatment of MOH to provide valuable evidence for clinical decision-making.

## 2 Methods

### 2.1 Study design

This systematic review was registered in PROSPERO (CRD42022315845). We included randomized controlled trials (RCTs) and cohort studies on the treatment of MOH using BoNTA. We also extracted reviews, case reports, and letters.

### 2.2 Inclusion criteria

We included studies that described participants as medication overuse headache or chronic migraine with medication overuse, but only if we were able to extract data.

### 2.3 Participants

We included participants aged 18 years or older, regardless of sex, race, social and economic status, profession, or residential location. The diagnostic criteria for MOH were adopted from any acceptable definition of MOH, including those in the second edition of the International Classification of Headache Disorders (ICHD-2) (14), ICHD – 3β (15), or ICHD-3 (1).

### 2.4 Interventions

The intervention was intramuscular injection of BoNTA.

### 2.5 Comparisons

We compared BoNTA injection with placebo therapy or other active prophylactic treatments. Co-interventions were accepted if administered to the patients in all the comparison groups.

### 2.6 Outcome measures

The research outcomes included reduction in the number of headache days per month, reduction in days of acute medication intake per month, changes in Migraine Disability Assessment questionnaire (MIDAS) scores, changes in Headache Impact Test (HIT-6) scores, and adverse events.

#### 2.6.1 Primary outcomes

The primary efficacy variable was reduction in the number of headache days per month.

#### 2.6.2 Secondary outcomes

Reduction in days of acute medication intake per month, changes in MIDAS and HIT-6 scores, and adverse events.

### 2.7 Search strategies

**Figure 1:**
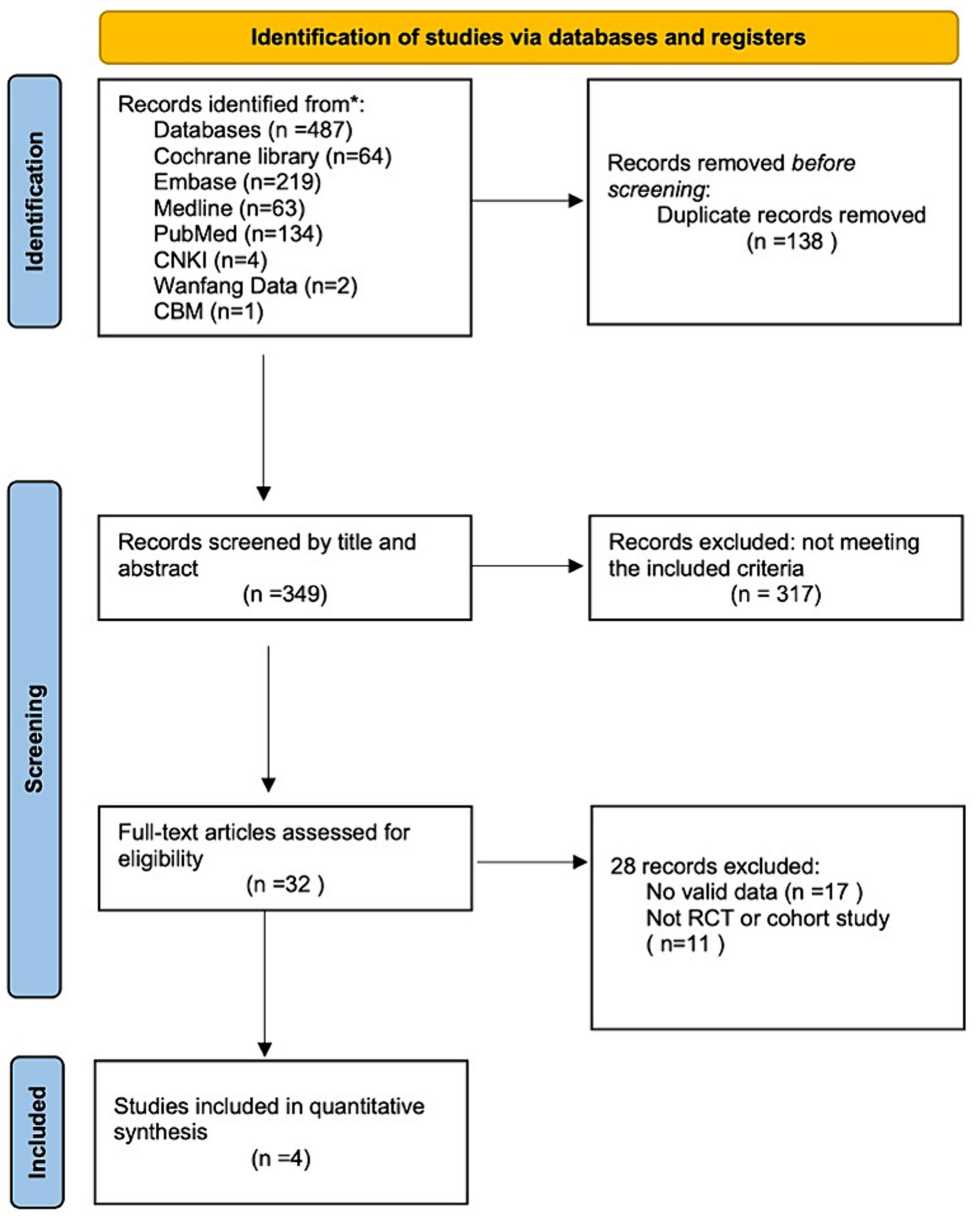
The selection process.

We followed the Preferred Reporting Items for Systematic Reviews and Meta-Analyses guidelines in this systematic review (16). The article search was conducted in March 2024. We searched four English online databases—Cochrane Library, Embase, Medline, and PubMed—and three Chinese electronic databases—China National Knowledge Infrastructure, Wanfang data, and Chinese BioMedical Literature Database—for studies on the treatment of MOH using BoNTA published between January 2002 and March 2024 (Figure 1). The publication type was restricted to articles on RCTs and cohort studies. To prevent omissions, the references from each article were also retrieved. We contacted authors for relevant data where necessary. The search was conducted using Medical Subject Heading terms and keywords. The search terms used and their relative variants are as follows: headache disorders, secondary, analgesic overuse headache, medication overuse headache, botulinum toxins, type A, botulinum toxin A, onabotulinumtoxinA, Botox, and Dysport.

### 2.8 Study selection, data collection, and management

Endnote 20 software was used to screen and eliminate duplicate studies. Two qualified investigators (Hui Lang and Cheng Peng) independently assessed the eligibility of the identified studies. Discrepancies were resolved through discussions with a third person (Ning Chen). The full text of each article was reviewed to ensure that the study met the requirements for primary and secondary outcomes. The review group created a unified data extraction table. A Microsoft Excel spreadsheet was used to extract all relevant data. The headings of the extraction table are as follows: (1) Basic information: study title, author’s name, publication date, and country; (2) Eligible data: diagnostic, inclusion, and exclusion criteria; (3) Intervention data: the number of patients allocated to the BoNTA and placebo groups, and the dose and duration of BoNTA treatment; and (4) Outcome measures: primary outcome, secondary outcome, adverse events, and measurement methods. During the entire process of data extraction, all discrepancies were resolved through discussion with a third reviewer.

### 2.9 Assessment of risk of bias

We used the Cochrane risk-of-bias tool for randomized trials to assess the risks of bias in the included RCTs, and used the Newcastle–Ottawa Scale to assess the cohort studies. Two reviewers independently used the Review Manager 5.4 software to assess the risks of bias in included studies in the following domains: random sequence generation, allocation concealment, blinding of participants and personnel, blinding of outcome assessment, incomplete outcome data, and selective reporting. The Newcastle–Ottawa Scale for cohort studies was used to assess the risks of bias in three domains: selection, comparability, and outcome.

### 2.10 Assessment of heterogeneity

Review Manager 5.4 software was used to test for heterogeneity, and the *I*^2^ index was used to estimate heterogeneity. *I*^2^ <50% indicates acceptable heterogeneity. The fixed effects model is used for the assessment of studies with low heterogeneity, whereas the random effects model is used for the assessment of studies with high heterogeneity.

## 3 Results

### 3.1 General characteristics of the included studies

A total of 487 potentially eligible articles were identified in the database search. Of these, 349 articles were retrieved after removing 138 duplicates. After reading the titles and abstracts of the remaining articles, 317 were excluded because they did not meet the inclusion criteria. The full texts of the remaining 32 articles were evaluated. Of these, 28 articles were excluded due to lack of extractable data (*n* = 17) or because they were reports of studies other than cohort studies or RCTs (*N* = 11). Finally, four RCTs were included in this systematic review, and no cohort/observational studies were included (13, 17–19).

A total of 1,259 participants with MOH, including 622 participants treated with BoNTA, 607 treated with a placebo, and 30 treated with topiramate, were included in the four RCTs. Three of the included studies (13, 17, 18) were randomized, double-blind, placebo-controlled clinical trials with a four-week baseline screening period. One study (19) did not include details of the method of randomization applied and did not follow the principle of blinding. Two of the studies (13, 17) had a 12-week double-blind phase, and the other two (18, 19) had a 24-week double-blind phase. It was worth noting that the population of studies by Pijpers et al. (13) and Sandrini et al. (17) conducted the randomization procedure in a homogeneous cohort exclusively suffering from MOH. However, the study by Silberstein SD et al. (18) was a post-hoc analysis of the Phase III Research Evaluating Migraine Prophylaxis Therapy (PREEMPT) that MOH subgroup was not independently randomized.

### 3.2 Risks of bias in the included studies

**Figure 2:**
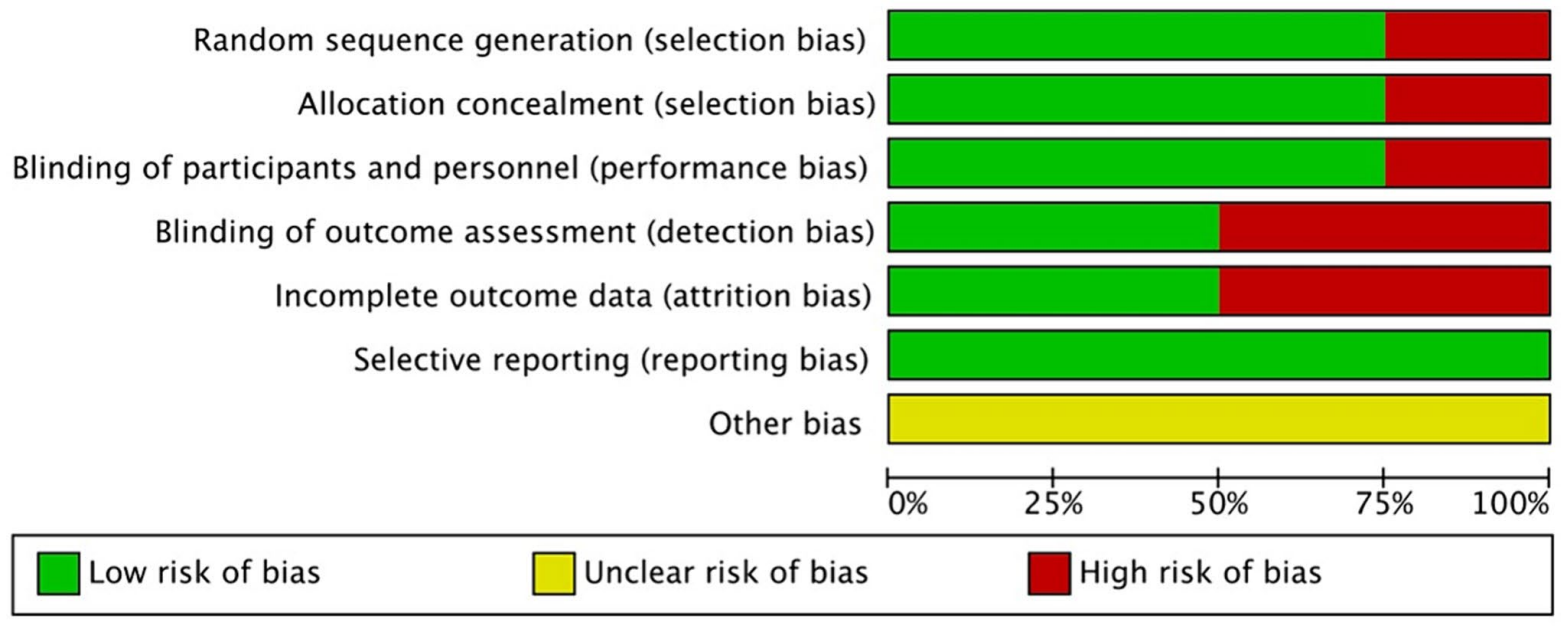
Risk of bias graph- review authors’ judgments about each risk of bias item for each included study.

**Figure 3:**
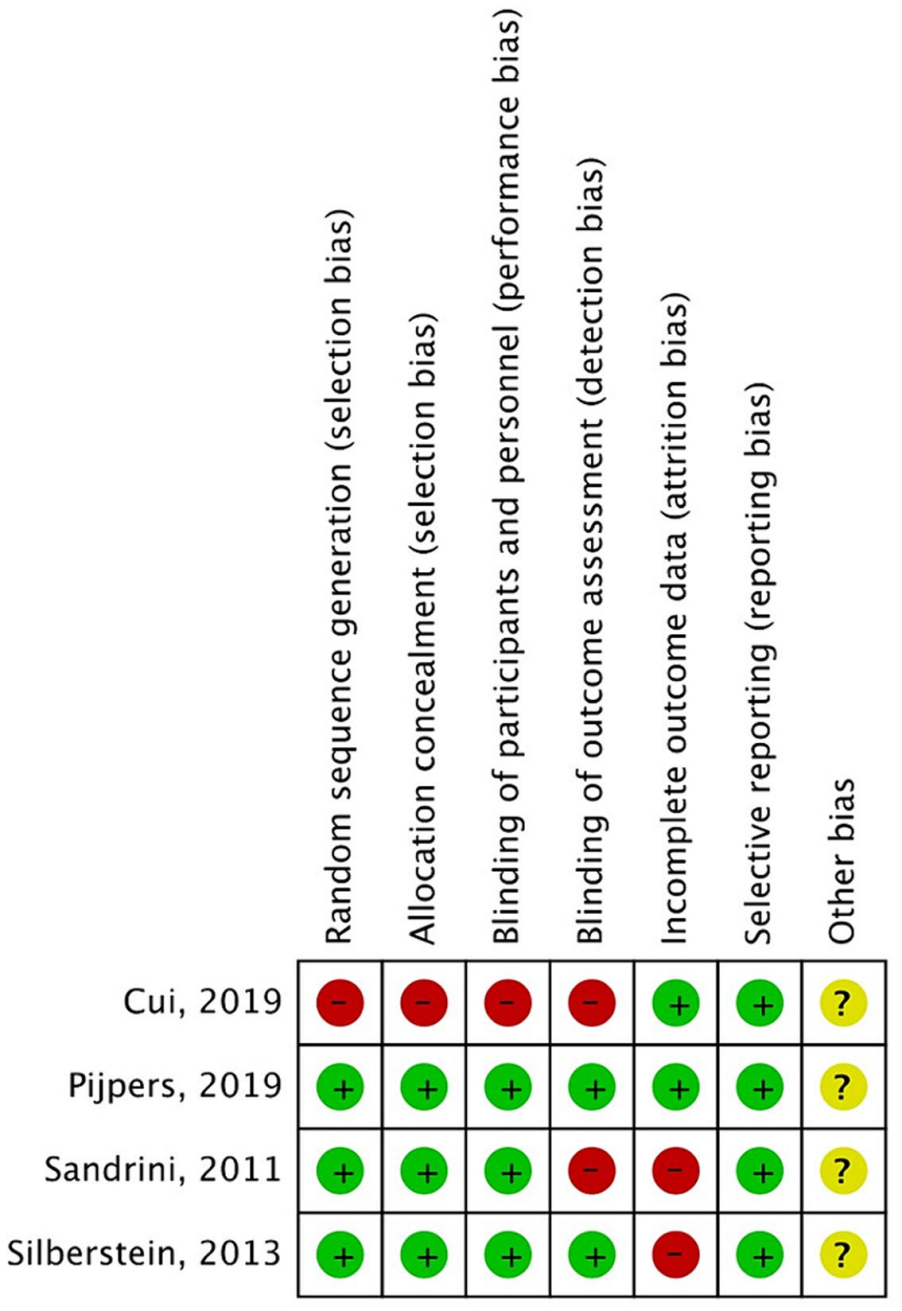
Risk of bias summary.

The Cochrane risk-of-bias tool was used to analyze the risks of bias in the included RCTs. Overall, there was a small variation in the risks of bias in the four studies (Figures 2, 3). Most of the studies had low risks of bias in the random sequence generation, allocation concealment, and selective reporting domains. One study (19) was considered to have a high risk of bias in random sequence generation, allocation concealment, blinding of participants and personnel, and blinding of outcome assessment because it did not include details of the exact method of randomization used and did not follow the principle of the blinding method. A proportion of studies were judged to have a high risk of bias for incomplete reporting because the number of participants included in the studies was small, and even a few loss-to-follow-up cases resulted in a high loss-to-follow-up rate. The studies with low, high, and unclear risks of bias in each domain are shown in Figure 2. The risks of bias in each study are shown in Figure 3.

### 3.3 Effects of intervention

#### 3.3.1 Primary outcome

##### 3.3.1.1 Comparison of the reduction in headache days per month in patients with MOH and controls

**Figure 4:**
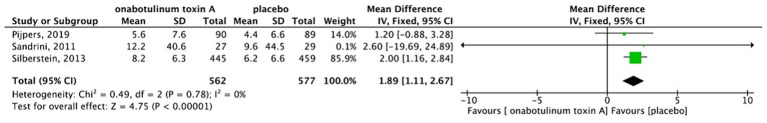
Forest plot of comparison in the reduction in headache days/month.

**Figure 5:**
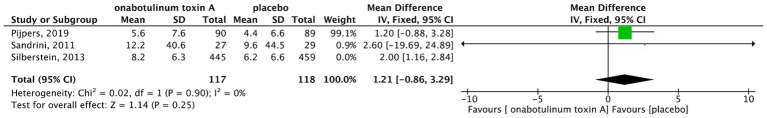
Forest plot of comparison in the reduction in headache days/month with sensitivity analysis.

There was no heterogeneity among three studies (13, 17, 18). Thus, a fixed-effects model was used. The results showed that patients with MOH treated using BoNTA had an average of 1.89 fewer headache days per month than the placebo group (MD, 1.89; 95% CI, 1.11–2.67; *I*^2^ = 0%; *p* < 0.001) (Figure 4). The study by Cui et al. (19) indicated that compared with topiramate, BoNTA significantly reduced the number of headache days per month in patients with MOH. Considering the randomization procedure of the study by Silberstein SD et al. (18), we performed a sensitivity analysis excluding the PREEMPT trials (Figure 5). The results showed that there was no difference in headache days between BoNTA and placebo groups. Egger’s test showed that there was no publication bias in our study (*p* = 0.109).

#### 3.3.2 Secondary outcomes

##### 3.3.2.1 Reduction in days of acute medication intake per month

**Figure 6:**
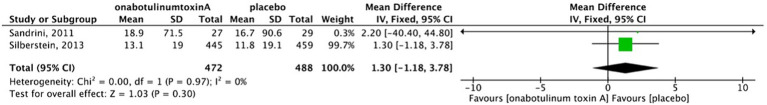
Forest plot of comparison- BoNTA versus placebo in reduction in days/month with acute medication intake.

There was no heterogeneity between two studies (17, 18); thus, we used a fixed-effects model to calculate the pooled effect size. The forest plot showed that there was no significant reduction in the number of days of acute medication intake per month after BoNTA therapy (MD, 1.3; 95%CI, −1.18-3.78; *I*^2^ = 0%; *p* = 0.30) (Figure 6). The study conducted by Cui et al. showed that BoNTA significantly reduced the frequency of acute medication intake compared topiramate (*p* = 0.027).

##### 3.3.2.2 Reduction in MIDAS scores

**Figure 7:**
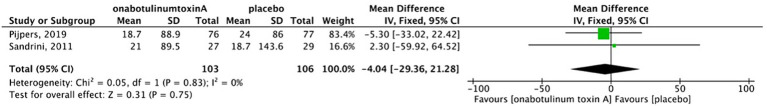
Forest plot of comparison- BoNTA versus placebo in reduction in MIDAS scores.

There was no heterogeneity between two studies (13, 17); thus, a fixed-effects model was used for analysis. The results indicate that there was no difference in MIDAS scores between the BoNTA and placebo group (MD, −4.04; 95%CI, −29.36-21.28; *I*^2^ = 0%; *p* = 0.75) (Figure 7). In the study by Cui et al. BoNTA more markedly reduced MIDAS scores compared with topiramate (*p* = 0.006).

##### 3.3.2.3 Reduction in HIT-6 scores

**Figure 8:**
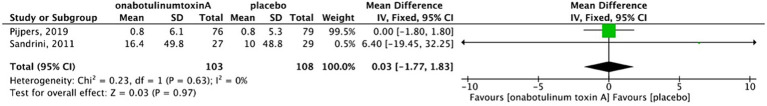
Forest plot of comparison- BoNTA versus placebo in reduction in HIT-6 scores.

There was no heterogeneity between two studies (13, 17); therefore, a fixed-effects model was used for analysis. The graph showed that there was no difference in HIT-6 scores between BoNTA and placebo group (MD, 0.03; 95%CI, −1.77-1.83; *I*^2^ = 0%; *p* = 0.97) (Figure 8).

### 3.4 Safety outcomes

**Figure 9:**
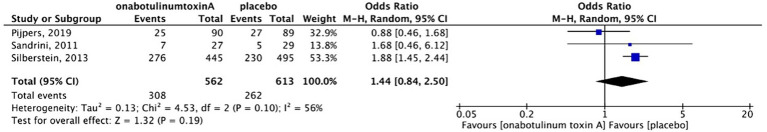
Forest plot of safety outcomes without sensitivity analysis.

**Figure 10:**
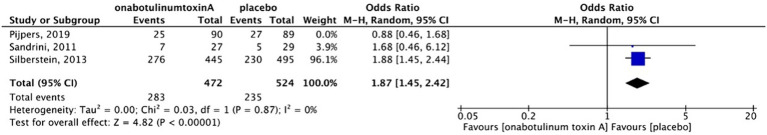
Forest plot of safety outcomes with sensitivity analysis.

Adverse events, including pain at the sites of injection, muscular weakness, and ptosis, were reported in three placebo-controlled studies. These adverse events were mild-to-moderate and temporary. Serious adverse events were not observed. Sensitivity analysis was performed because in Pijper’s study (13), the participants in the placebo group received a small dose (17.5 units) of BoNTA to ensure that the patients were blinded to the procedures. After excluding the study based on the sensitivity analysis (Figures 9, 10), we found that patients treated with BoNTA were more susceptible to adverse events than those in the placebo group (OR, 1.87; 95%CI, 1.45–2.42; *I*^2^ = 0%; *p* < 0.001). Cui et al. reported adverse events in both the BoNTA and topiramate groups. However, 40% of participants in the BoNTA group experienced adverse events, including pain at the sites of injection, local asthenia, mild swelling, and muscular pain, whereas 86.7% of the participants in the topiramate group experienced adverse events, including dizziness, drowsiness, and limb numbness.

## 4 Discussion

### 4.1 Summary of main results

In the present systematic review, we analyzed four RCTs with a total of 1,259 patients with MOH, including 622 participants treated with BoNTA, 607 treated with a placebo, and 30 treated with topiramate. The study by Cui et al. (19) was published in China in 2019, and included participants who visited the headache clinic of the Department of Neurology of Shandong Provincial Hospital from March 2014 to February 2016. However, the authors did not describe the method of randomization used and did not follow the principle of blinding. In addition, a survey (20) published in 2021 in China revealed that clinical research papers in published Chinese medical journals have low quality and a high risk of bias. BoNTA was effective in reducing the frequency of headaches in the three placebo-controlled studies included in this systematic review. However, there was no significant difference in secondary outcomes between BoNTA and placebo groups. Data on the safety profile of BoNTA revealed that patients treated with BoNTA were more likely to experience adverse events than those treated with a placebo (Table 1).

**Table 1:** Characteristics of the included studies.

Author, year	Country	MOH definition	Sample size		Follow-up	Baseline number of headache days per month			Inclusion criteria
			BoNTA	Control group		BoNTA	Control group	*p* value	
Sandrini, 2011	Italy	The 2nd edition of the International Headache Society’s International Classification of Headache Disorders (ICHDII, IHS 2004)	27	29 (placebo)	12 weeks	24.2	25.5 (placebo)	0.209	Eligible patients were men or women aged 18–65 years with a history of headache that fulfilled the diagnostic criteria for migraine without aura as the primary headache plus medication-overuse headache, with *≥*15 headache days every 4 weeks over the past 3 months, and with each headache day consisting of *≥*4 h of continuous headache with mostly migraine features.
Pijpers, 2019	Netherlands	Headache Classification Committee of the International Headache Society, 2013	90	89 (placebo)	12 weeks	21.7	21.0 (placebo)	/	Patients aged 18–65 years who were able to comply with the study protocol and provided written informed consent were eligible. Consecutive patients with chronic migraine and medication overuse headache (Headache Classification Committee of the International Headache Society, 2013). Diagnoses were established after consultation with headache experts and confirmed by based on data from a 4-week baseline headache diary.
Silberstein, 2013	United States	The 2nd edition of the International Headache Society’s International Classification of Headache Disorders (ICHDII, IHS 2004)	445	459 (placebo)	24 weeks	20.1	19.8 (placebo)	0.278	Men or women aged 18 to 65 years old with a history of migraine as defined in the second edition of the International Classification of Headache Disorders (ICHD-II) Section 1, Migraine. Eligible patients had headache that occurred on ≥15 days in 4 weeks, with each day consisting of ≥4 h of continuous headache, and ≥ 50% of the baseline headache days being migraine or probable migraine days (referred to as migraine days) and ≥ 4 distinct headache episodes lasting ≥4 h each month.
Cui, 2019	China	The International Classification of Headache Disorders 3rd beta edition, (ICHD-III)	30	30 (topiramate)	24 weeks	20.6	21.1 (topiramate)	/	(1) Participants with MOH met the diagnostic criteria of the ICHD-IIIβ for CM and refractory migraine proposed by the American Headache Society. Participants with MOH met the diagnostic criteria of the ICHD-IIIβ. (2) After the adjustment of inducing factors and lifestyle, adequate acute and preventive treatment, headache still significantly affected participants’ daily activities and quality of life. (3) No topiramate medication history. (4) History of drug withdrawal failure. (5) No other drug trials within 6 months. The individuals voluntarily participated in the clinical trial and had no obvious language communication disorder. (6) The participants or their family members voluntarily signed informed consent forms. (7) The study was approved by the hospital ethics committee.

BoNTA can be used to alleviate CM in clinical practice. PREEMPT1 and PREEMPT2 (21, 22) studies showed that compared with placebo, BoNTA significantly reduces monthly frequency of headache, headache intensity, and medication intake with few treatment-related adverse events in individuals with CM (21, 22). The results of our study also indicated that BoNTA treatment has a positive effect on MOH, and can reduce the frequency of headache. This may be related to the botulinum toxin inhibiting neurogenic inflammation and reducing the peripheral sensitivity of nociceptive nerve fibers, thereby relieving headaches (10, 11).

The results of this study showed that BoNTA did not reduce MIDAS or HIT-6 scores in patients with MOH. This may be attributable to a few factors. First, the RCT conducted by Silberstein (18) was a study of patients with MOH included in the PREEMPT study, which excluded patients with daily headache or comorbid depression (21, 22), whereas the studies by Pijpers (13) and Sandrini (17) included these patients. This may have affected the evaluation of headache, lowering the baseline HIT-6 and MIDAS scores in the PREEMPT study, thereby affecting the change in HIT-6 and MIDAS scores in patients with MOH treated using BoNTA. Second, Silberstein’s study was conducted using the diagnostic criteria of ICHD-2 (14), which are less extensive than those of the ICHD-3 (1, 23). Therefore, some patients with MOH may have been omitted during the screening process. Third, in the studies by Pijpers and Sandrini, the double-blind phase was 12 weeks. However, it is possible that some patients with MOH may not show any treatment response until 12 weeks later. In a two-year prospective study, researchers found that BoNTA could significantly decrease the mean HIT-6 score (pre-treatment, 69.4 ± 4.9; post-treatment, 52 ± 5.6) during treatment (24). Finally, the sample size of Sandrini’s study (BoNTA = 33, placebo = 35) was small, and this may have affected the results of this review.

The adverse events of BoNTA reported in the included studies were mild or moderate. They mainly included pain and a small hematoma at the site of injection, ptosis, and muscle weakness. The pain and the small hematoma were caused by the injection. The ptosis and muscle weakness may be related to the inhibition of acetylcholine release from peripheral nerve cells to the neuromuscular junction, thereby relaxing muscles (25). All the reported adverse events were temporary. No serious adverse events during or after BoNTA therapy were reported in any of the three placebo-controlled studies or in the topiramate-controlled study.

### 4.2 Quality of the evidence

All three placebo-controlled trials were randomized, double-blind, and placebo-controlled. All three studies included clear descriptions of the generation of random sequences, concealment of allocation methods, blinding of participants and personnel, and selective reporting. However, two of the studies (17, 18) had a high risk of bias for incomplete outcome data because of the high dropout rate in the studies (>5%). All the studies had an unclear risk of bias owing to other biases. The topiramate-controlled study had a high risk of bias in random sequence generation, allocation concealment, blinding of participants and personnel, and blinding of outcome assessment. The authors did not describe the method of randomization and did not follow the blinding principle.

### 4.3 Potential biases in the review process and applicability of evidence

We searched four English electronic databases and three Chinese electronic databases for relevant literature. We also contacted the investigators to acquire detailed data on the studies but failed to obtain additional information. This may have led us to omission of some studies. Two studies included in our review had small sample sizes, and two were conducted using the ICHD-2 diagnostic criteria for MOH. Owing to the lack of standardization of the experimental methods, the validity of our conclusions was affected. Therefore, the applicability of the findings of this review is undetermined.

### 4.4 Agreements and disagreements with other studies or reviews

During our database search, we found a systematic review and meta-analysis (26) on BoNTA for the treatment of patients with chronic migraine and medication overuse headache, which was published in April 2023. The study, which was conducted by Giri et al., concluded that BoNTA reduces the frequency of headache, a finding that is similar to that of the present study. Furthermore, we investigated acute medication intake, MIDAS scores, and HIT-6 scores to assess the efficacy of BoNTA for MOH. Chiang et al. published a systematic review of MOH treatment, which indicated that BoNTA is an effective preventive treatment for MOH, a finding that is also similar to the results of the present study (7). The systematic review by Chiang et al. also indicated that administration of BoNTA therapy without early discontinuation significantly reduces headache days, whereas early discontinuation of BoNTA therapy significantly reduces acute medication intake (7). In addition, a two-year prospective study demonstrated that BoNTA 195U is significantly more effective than 155U in reducing the mean number of headache days, medication intake days, and HIT-6 score (27). However, the study indicated that the difference in treatment-related adverse events between BoNTA 195U and 155U is not statistically significant, and that the adverse events are mild to moderate, lasting for approximately 1 week (27). Notably, Pijpers (13) reported that BoNTA does not significantly reduce the number of headache days per month in patients with MOH compared with placebo, and does not provide additional benefit to withdrawal therapy. This may be due to the relatively short duration of the 12-week study and its small sample size. Overall, the efficacy and safety of BoNTA for treatment of MOH are still controversial. Therefore, larger randomized controlled trials are required to clarify the efficacy and safety of BoNTA for treatment of MOH.

### 4.5 Limitations

There are limitations in our study. RCTs minimize selection bias and other potential confounding factors through random allocation of participants to treatment and control groups, ensuring reliable results that directly assess the effects of interventions (28). Cohort Studies excel in observing long-term associations between exposure factors and outcomes. They provide valuable insights into drug effects and side effects (28). In our systematic review, we had originally planned to include both RCTs and cohort studies to help investigate the efficacy and safety of BoNTA in MOH. However, we ultimately included only four RCTs, and no cohort/observational studies. It’s important to note that the study by Silberstein SD et al. (18) is a post-hoc analysis of the PREEMPT1 and PREEMPT2 trials (21, 22), which were randomized into BoNTA and placebo group on the basis of CM, and the MOH subgroup was not randomized independently. In contrast, a homogeneous cohort with only MOH was used for randomization in the studies by Pijpers et al. (13) and Sandrini et al. (17). The PREEMPT trial included a larger population with more statistical power, and sensitivity analysis showed no effect of BoNTA on headache frequency in MOH. Our findings need more clinical trials.

## 5 Conclusion

This study showed that BoNTA can be used for the treatment of patients with MOH because it significantly reduces the frequency of headache. However, considering that the trials included in this review are underpowered, the results are far from robust. Further large-scale research on the efficacy and safety of BoNTA is urgently needed.

## Data Availability

The original contributions presented in the study are included in the article/supplementary material, further inquiries can be directed to the corresponding author.
